# Relationship Between the Onset of Ménière's Disease and Sympathetic Hyperactivity

**DOI:** 10.3389/fneur.2022.804777

**Published:** 2022-03-17

**Authors:** Masanori Ishii, Gail Ishiyama, Akira Ishiyama, Yujin Kato, Fumihiro Mochizuki, Yusuke Ito

**Affiliations:** ^1^Department of Otorhinolaryngology, Japan Community Health Care Organization (JCHO) Tokyo Shinjuku Medical Center, Tokyo, Japan; ^2^Department of Otorhinolaryngology and Head & Neck Surgery, The Jikei University School of Medicine, Tokyo, Japan; ^3^Department of Neurology, David Geffen School of Medicine at UCLA, Los Angeles, CA, United States; ^4^Department of Head & Neck Surgery, David Geffen School of Medicine at UCLA, Los Angeles, CA, United States; ^5^Department of Otorhinolaryngology, St. Marianna University School of Medicine, Kanagawa, Japan

**Keywords:** central autonomic network, Ménière's disease, sympathetic nervous system, over stress, shear stress, vascular permeability, TRPV4, shimoyake

## Abstract

**Objective:**

The pathogenesis of Ménière's disease is still largely unknown; however, it is known to be strongly associated with stress. Excessive stress can cause hyperactivity of the sympathetic autonomic nervous system. With the aim of understanding changes in sympathetic hyperactivity before and after Ménière's disease, we compared autonomic nervous function in patients in a stable phase of Ménière's disease and that in healthy adults. We also gathered data over about 10 years on autonomic nervous function immediately before a Ménière's attack.

**Study Design:**

Prospective study.

**Patients:**

Autonomic nervous function was analyzed in 129 patients in a stable phase of Ménière's disease 31 healthy adult volunteers. In nine patients, autonomic nervous function was also measured immediately before and after treatment of a vertigo attack.

**Main Outcome Measure:**

Power spectrum analysis of heart rate variability (HRV) of EEG/ECG and an infrared electronic pupillometer were used. Sympathetic and parasympathetic nervous function was measured.

**Results:**

There were no statistically significant differences in autonomic nervous function determined by HRV and electronic pupillometry between patients in a stable phase of Ménière's disease and healthy adults. Sympathetic function as measured by electronic pupillometry parameters VD and T5 showed no difference between the affected and unaffected sides in the baseline data measured in the stable phase (VD: affected side is 31.02 ± 6.16 mm/sec, unaffected side is 29.25 ± 5.73 mm/sec; T5: affected side is 3.37 ± 0.43 msec, unaffected side is 3.25 ± 0.39 msec). In contrast, all nine patients whose HRV data had been obtained just before an attack showed marked suppression of the parasympathetic nervous system and activation of the sympathetic nervous system. Electronic pupillometry also revealed an overactivation of the sympathetic nervous system on the affected side, just before the attacks. Analysis of sequential changes after the onset of an attack revealed that overactivation on the affected side was reduced after treatment, and no difference between affected and unaffected sides was observed 3 days after treatment.

**Conclusion:**

Detailed analysis of autonomic nervous function showed that immediately before an attack of Ménière's disease, the sympathetic nervous system on the affected side was strongly overactivated.

## Introduction

Although it has been almost 160 years since Ménière's disease was first described (1), it is still a disease whose cause is unknown. The membrane rupture theory (2) to explain the pathogenesis of Ménière's disease is well-known; however, that theory cannot explain all aspects of the disease. For example, why does stress (3, 4) trigger the onset of attacks? Why does endolymphatic hydrops occur? Why is low-tone sensorineural hearing loss predominant? Why do both cochlear and vestibular symptoms occur? Why does irritative nystagmus occur during the acute phase, followed by paralytic nystagmus? Why do symptoms worsen and resolve rapidly by the minute or by the hour? Ménière's disease is full of mystery.

There are many potential causes of Ménière's disease, including allergy (5), infection, hormonal disorders, genetic disorders, autoimmune diseases (6), and psychological load (7); however, at present there is no definitive theory to explain the pathogenesis of the disease (8).

Although involvement of the autonomic nervous system has been suggested, only a few studies have been performed to examine its association with Meniere's attacks using electronic pupillometry (9). We report here that we were able to obtain convincing evidence by analyzing autonomic function.

## Subjects and Methods

In this prospective study, we examined 129 patients with a history of active or stable Ménière's disease (32 men and 97 women; mean age ± SD, 44.7 ± 19.3 years) and 31 healthy adult volunteers (nine men and 22 women; mean age ± SD, 38.8 ± 5.3 years) after obtaining their written informed consent.

The patients with Ménière's disease were those who had presented to our hospital from 1999 to 2021. Patients were defined to be in a stable phase of Ménière's disease if they met the criteria of definite Ménière's disease in the diagnostic criteria by the American Academy of Otolaryngology-Head and Neck Surgery (10), had had no vertigo attacks for more than 3 months, and had discontinued medication. The control group included healthy volunteers without a past history of inner or middle ear disorders or any underlying diseases, such as hypertension, diabetes mellitus, or autonomic dysfunction.

Detailed inclusion and exclusion criteria were as follows. Inclusion criteria: (1). age between ≥20 and <60 at the time of consent; (2). patients who had been followed up for at least 1 years after definitive diagnosis of Ménière's disease based on the American Academy of Otolaryngology-Head and Neck Surgery diagnostic criteria for Ménière's disease (10); and (3). patients who gave consent after thorough explanation and understanding of this clinical trial. Exclusion criteria: (1). persons who did not have or had not had any inner or middle ear disorders; (2). persons who had a past history or complication of malignant tumor (those who had not relapsed for more than 5 years were eligible for inclusion); (3). persons diagnosed as having sleep apnea, sleep disorder, dementia, or psychiatric disorder receiving treatment; (4). persons who had complications that would have a significant impact on the assessment of autonomic function, including diabetes mellitus, Parkinson's disease, and autonomic dysfunction; (5). persons who had eye diseases under treatment with eye drops such as mydriatics or ophthalmic anesthetics, including allergic conjunctivitis, keratitis, diabetic retinopathy, extraocular inflammation, infectious diseases, and severe dry eye; (6). persons who had stress-related disorders, such as gastro-esophageal reflux disease (GERD), severe tension headache, severe stiff neck and shoulders requiring treatment, and asthma; (7). persons with hypertension undergoing oral treatment; (8). women who were pregnant, lactating (including women who would stop lactation), or those who wished to become pregnant during the trial period; and (9). persons who were otherwise considered ineligible as participants of this clinical trial by the investigator or sub investigators.

Autonomic nervous system function was measured with an EEG/ECG monitor (MWM-20; GMS Co., Ltd., Tokyo, Japan), with real-time power spectrum analysis of heart rate variability (HRV) performed with Mem Calc software (GMS Co., Ltd.) on a Windows 10 computer linked to the monitor via Bluetooth. High-frequency spectral power (HF, 0.15–0.50 Hz) containing parasympathetic nerve components, low-frequency spectral power (LF, 0.05–0.15 Hz) containing both sympathetic and parasympathetic components, and heart rate were measured over time (11, 12).

**Figure 1:**
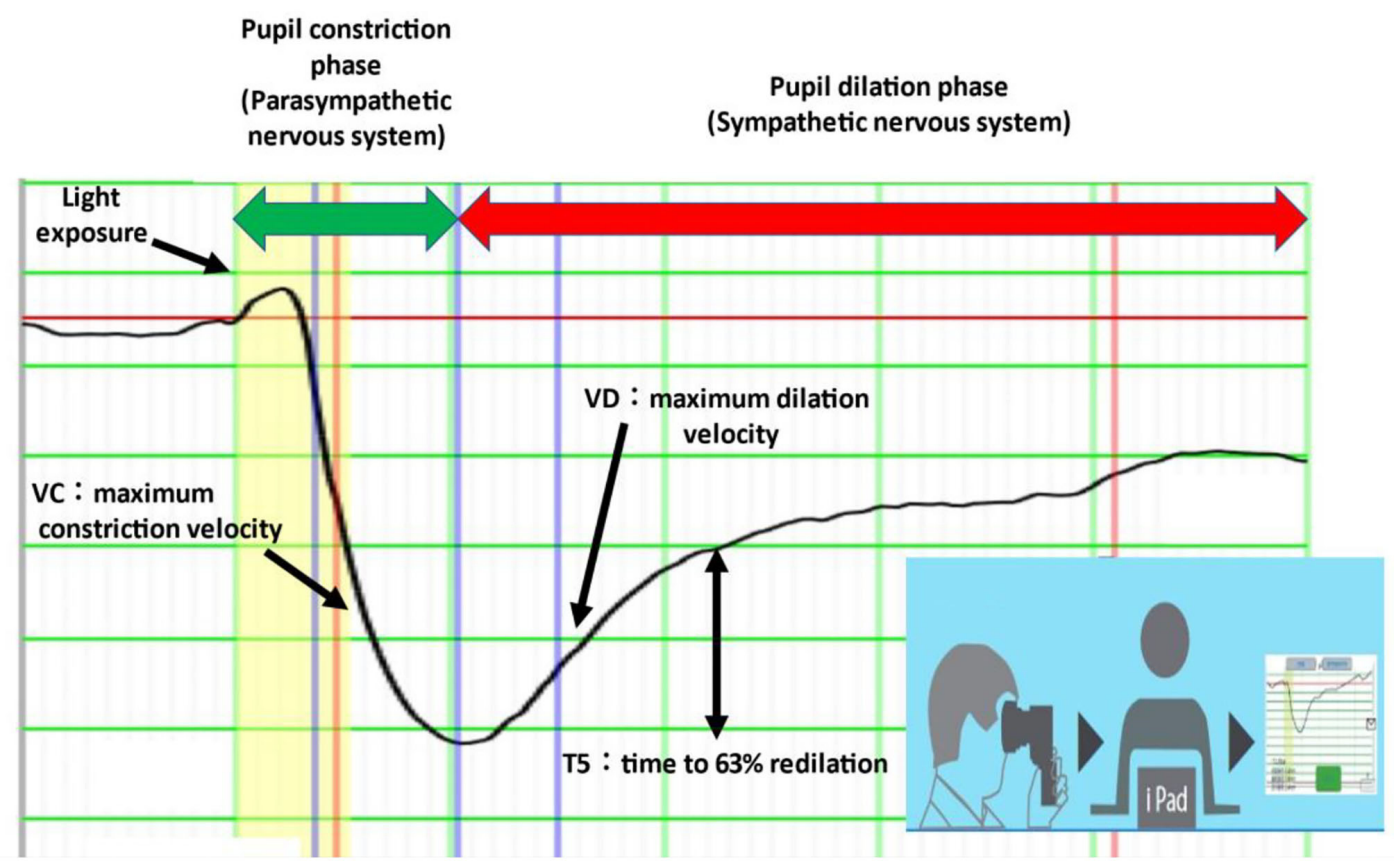
Electronic pupillometry. VD and T5 represent sympathetic activity and VC represents parasympathetic activity. The measuring device is portable and can be connected to an iPad via Wi-Fi. Each measurement takes less than 1 min.

Electronic pupillometry was also performed using a portable measuring device (DK-100; Scalar Corporation, Tokyo, Japan). This device uses an infrared CCD video camera to capture changes in pupil size that occur with the pupillary light reflex and sends data via Wi-Fi to an iPad for analysis (Figure 1) (13, 14). The pupillary light reflex can be used to assess autonomic nervous system function: the pupil constriction phase represents parasympathetic function and the pupil dilation phase represents sympathetic function. VC (maximum constriction velocity) is a parameter for parasympathetic function, and VD (maximum dilation velocity), and T5 (time to 63% redilation) are parameters for sympathetic function (15).

Acquisition of baseline data was performed on Saturdays or Sundays at times between 9 a.m. and 4 p.m. when the hospital was very quiet. To allow autonomic nerves time to stabilize, measurements were performed more than 2 h after a meal. The same dark room and chair were always used for measurements. Subjects were asked to sit on the chair with both palms resting lightly on their thighs without leaning against the back of the chair.

HRV for power spectrum analysis was measured for 20 min while the subjects were at rest with their eyes closed. The mean values of HF and LF were calculated over 5 min—from 15 to 20 min after the initiation of the measurement—when values had stabilized. HF represents parasympathetic activity and LF represents a composite of sympathetic and parasympathetic activities (16). Pupillometry to measure VD and T5 (i.e., parameters of sympathetic function) and VC (i.e., a parameter of parasympathetic function) was performed immediately after the power spectrum measurement of HRV.

The patients whose baseline data had been obtained were instructed to visit the hospital as soon as possible if they experienced feelings of an oncoming attack (such as exacerbation of aural fullness, tinnitus, head heaviness, or stiff neck and shoulders).

Our hospital, the Japan Community Health Care Organization, Tokyo Shinjuku Medical Center, is located in the center of Tokyo where patients can easily visit and has a close relationship between patients and doctors. Such an environment enabled us to perform this study over more than 10 years.

The EEG/ECG device was fitted to the patient on arrival at the outpatient department which has offices and rooms for treatment, allowing measurements of autonomic nervous function (by HRV) to be taken before an attack. Electronic pupillometry required only 1 min or less for each measurement. Treatment was started immediately after measurements before and during an attack. After therapeutic infusion lasting approximately 30 min, measurement was performed again. Severe vertigo attacks were resolved in 30 min (by the end of the infusion) in all patients who received treatment.

Of a total of 589 measurements, including several attacks per patient, only nine measurements (1.5%) were able to be obtained immediately before an attack.

Meniere's disease is associated with a variety of co-occurring symptoms. We collected and analyzed data from a large number of patients for these as well. We analyzed the HRV just before the attack, as well as changes in autonomic nerve function using an electronic pupillometer just before the attack, just after the attack, just after treatment, and 3 days after treatment, and changes in co-occurring symptoms over the same time course.

These nine patients with Ménière's disease comprised three men and six women; mean age ± SD, 38.75 ± 7.98 years.

Statistical analysis of data was performed on a Windows 10 PC using add-on software (Statcel Ver. 4, Microsoft Excel) dedicated to statistical analysis.

## Results

**Figure 2:**
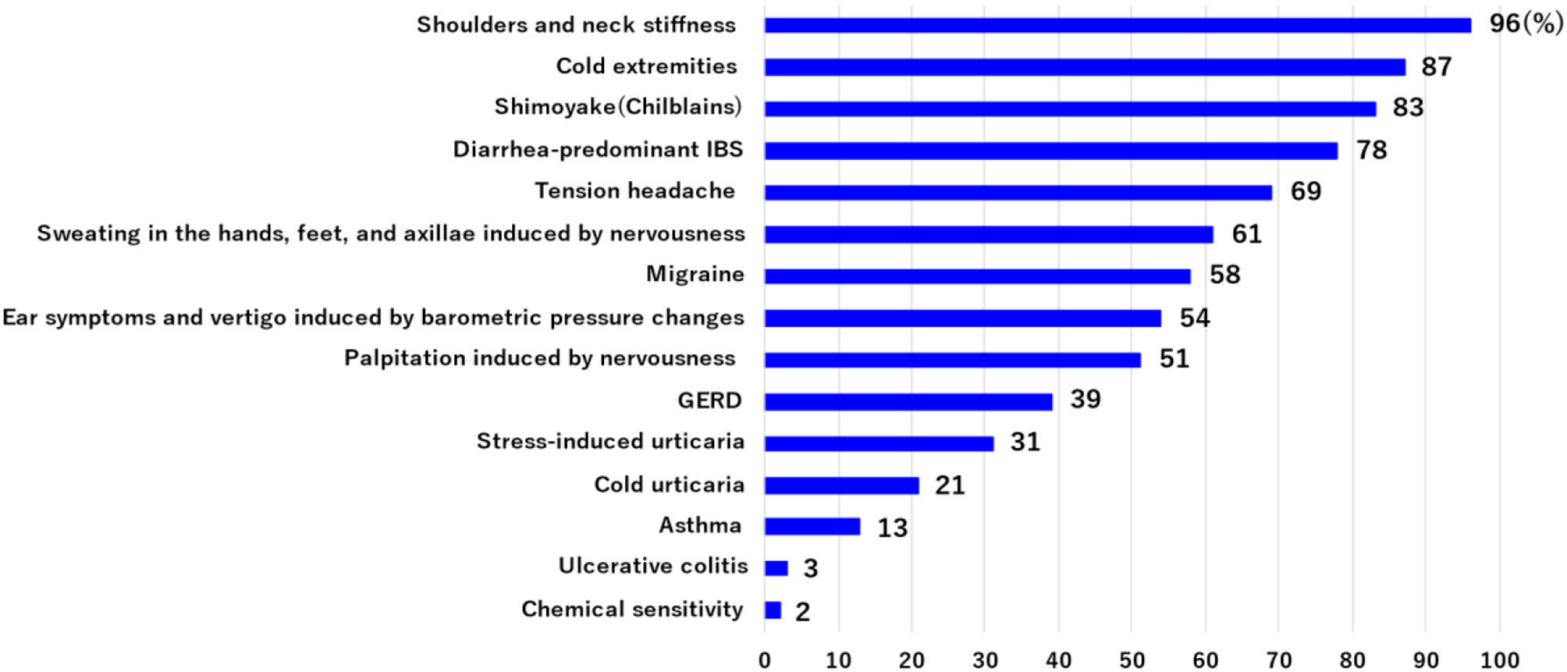
The incidence of co-occurring symptoms was evaluated in 211 patients with Ménière's disease who had had three or more vertigo attacks. These symptoms include multiple occurrences. Many of these symptoms are associated with stress and are accompanied by the excitation of sympathetic nerves.

Patients with Ménière's disease usually have a number of symptoms in addition to vertigo and hearing loss. We evaluated the incidence of such co-occurring symptoms in 211 patients with Ménière's disease who had had three or more vertigo attacks. The most frequent symptom (including multiple occurrence) was neck and shoulder stiffness (96%), followed by cold extremities (87%), *shimoyake* (chilblains) (83%), diarrhea-predominant irritable bowel syndrome (D-IBS) (73%), tension headache (69%), sweating in the hands, feet, and axillae induced by nervousness (61%), migraine (58%), ear symptoms and vertigo induced by barometric pressure changes (54%), palpitation induced by nervousness (51%), GERD (39%), stress-induced urticaria (cholinergic urticaria, physical urticaria) (31%), cold urticaria (21%), asthma (13%), ulcerative colitis (3%), and chemical sensitivity (2%) (Figure 2). Many of these symptoms are thought to be associated with stress and are accompanied by the excitation of sympathetic nerves.

To exclude the influence of age-related changes in autonomic nerves (17), we compared the preliminary data in a set of age-matched healthy adults and patients in a stable phase of Ménière's disease (range, 24–48 years; mean age, 36.2 years). No statistically significant difference was found in the HF and LF values obtained from HRV power spectrum analysis between healthy adults and patients in a stable phase of Ménière's disease (Table 1, upper table).

**Table 1:** HRV power spectrum analysis in 31 patients in a stable phase of Ménière's disease and 31 age-matched healthy adults (upper table), and HRV power spectrum analysis in a stable phase and just before an attack in nine patients whose autonomic function was able to be measured immediately before an attack (lower table).

	**Healthy adults** **(***n*** = 31)**	**Ménière's disease (stable phase) (***n*** = 31)**	***P*** **(Welch's ***t***-test)**
HF (msec^2^)	429.52 ± 201.09	391.29 ± 166.71	*P* = 0.429
LF (msec^2^)	449.06 ± 85.23	493.43 ± 124.07	*P* = 0.107
LF/HF	1.28 ± 0.649	1.52 ± 0.573	*P* = 0.212
Heart rate (bpm)	68.21 ± 5.88	69.13 ± 6.24	*P* = 0.551
**Analysis of Ménière's disease just before an attack (*****n*** **=** **9)**			
	**Stable phase before an attack**	**Immediately before an attack**	***P*** **(paired** ***t*****-test)**
HF (msec^2^)	466.66 ± 178.61	150.66 ± 77.43	*P* = 0.00039
LF (msec^2^)	545.21 ± 178.61	1347.22 ± 923.1	*P* = 0.00175
LF/HF	1.36 ± 0.80	9.69 ± 6.34	*P* = 0.00243
Heart rate (bpm)	70.56 ± 8.85	87.0 ± 8.73	*P* = 0.00103

However, just before an attack, the HF value which represents parasympathetic function was significantly lower than the HF value in the stable phase in all nine patients (*P* < 0.01) (Table 1, lower table). The LH value (*P* < 0.01) and heart rate (*P* < 0.01) representing sympathetic function were statistically significantly higher just before an attack than the values in the stable phase. This suggests that, just before an attack of Ménière's disease, the parasympathetic nervous system is markedly suppressed while the sympathetic nervous system is markedly activated, which results in further significant relative activation of the sympathetic nervous system.

The analysis of 31 patients in a stable phase of Ménière's disease and 31 age-matched healthy adults by electronic pupillometry showed that T5 representing sympathetic activity tended to be lower on the affected side of the patients than on the unaffected side and was lower in the patients than in the healthy adults, but there was no significant variation between subgroups. There was also no significant difference in VC and VD between the affected and unaffected sides of the patients or between the affected side of the patients and healthy adults (single-factor ANOVA) (Table 2).

**Table 2:** Electronic pupillometry analysis in 31 patients in a stable phase of Ménière's disease and 31 age-matched healthy adults.

			**Ménière's disease (Stable phase)**	**Ménière's disease (Stable phase)**	**Heathy adults**	**Heathy adults**
			**Affected side**	**Unaffected side**	**Right side**	**Left side**
VC (mm/sec)	Average		145.10	148.87	181.05	167.26
	S.D.		13.59	13.68	13.46	12.93
	*P*-value	0.719				
VD (mm/sec)	Average		34.45	32.84	34.71	34.77
	S.D.		5.94	4.98	6.45	5.18
	*P*-value	0.493				
T5 (msec)	Average		3.50	3.52	3.79	3.79
	S.D.		0.49	0.57	0.79	0.61
	*P*-value	0.098				

*There was no significant difference in VC, VD, and T5 between the affected side and the unaffected side of the patients or between the affected side of the patients and the healthy control group (single-factor ANOVA)*.

**Figure 3:**
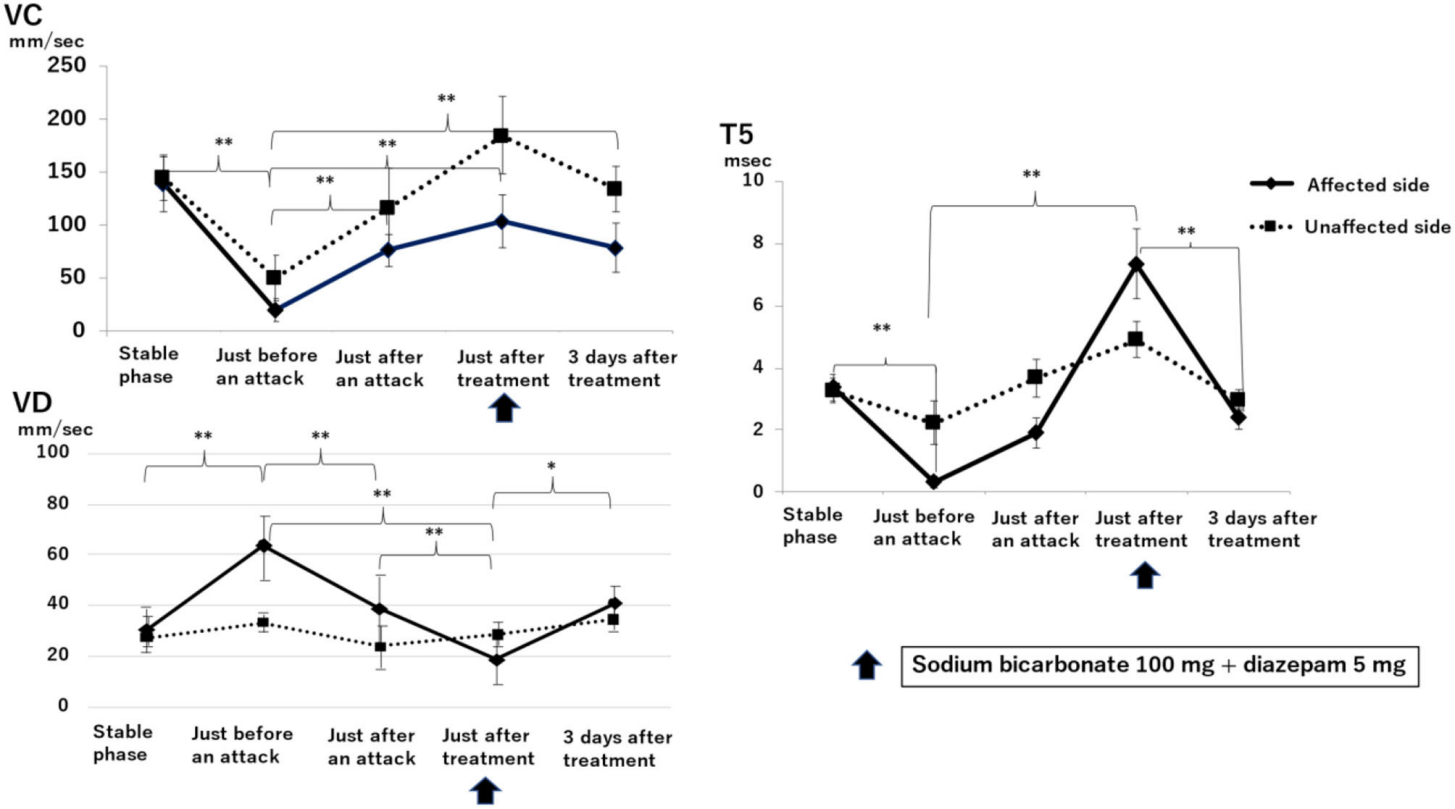
Sequential changes in the autonomic nervous system as measured by electronic pupillometry just before, just after, and 3 days after a Ménière's disease attack. Maximum constriction velocity, VC (mm/sec): larger values indicate a more activated parasympathetic nervous system. Maximum dilation velocity, VD (mm/sec): larger values indicate a more activated the sympathetic nervous system. T5 (msec): smaller values indicate a more activated sympathetic nervous system. An infusion of 100 mg sodium bicarbonate plus 5 mg diazepam was administered for its antiemetic effect once just after an attack. A significant reduction in parasympathetic activity and excitation of sympathetic activity was found on the affected side just before an attack. Just after an attack, parasympathetic activity on the affected side was activated, followed by the recovery of parasympathetic activity and suppression of sympathetic activity after treatment. Results with statistically significant differences by two-factor ANOVA and then Scheffe's F-test (multiple comparison test) are shown between curly brackets. **P < 0.01, *P < 0.05. Data are presented as mean ± standard deviation (*n* = 9).

Figure 3 shows the results of electronic pupillometry in the nine patients with Ménière's disease in the stable phase, just before an attack, just after an attack, just after treatment, and 3 days after treatment.

VC, a parameter for parasympathetic function, on the affected side just before an attack (19.00 ± 10.06 mm/sec) was significantly lower than that in the stable phase (138.25 ± 25.24 mm/sec) (*P* < 0.01, two-factor factorial ANOVA; Scheffe's *F*-test). VC was 75.65 ± 15.63 mm/sec just after an attack and increased to 103.45 ± 24.41 mm/sec at 30 min after infusion of sodium bicarbonate (100 mg) plus diazepam (5 mg). Three days after treatment, VC was 83.03 ± 44.11 mm/sec, significantly higher than that just before an attack (*P* < 0.01).

VD, a parameter for sympathetic function, on the affected side just before an attack (63.90 ± 12.73 mm/sec) was significantly higher than that in the stable phase (31.02 ± 6.16 mm/sec) *(P* < 0.01). VD decreased to 38.73 ± 13.90 mm/sec just after an attack and decreased further to 18.70 ± 8.88 mm/sec just after treatment, which is markedly lower than that just before an attack (*P* < 0.01). 3 days after treatment, VD increased to 40.70 ± 6.62 mm/sec, which is higher than that just after treatment (*P* < 0.05).

T5 is also a parameter for sympathetic function, and lower values indicate a more activated sympathetic function. T5 just before an attack on the affected side (0.35 ± 1.90 msec) was markedly lower than that in the stable phase (3.37 ± 0.43 msec) (*P* < 0.01). T5 increased to 1.92 ± 0.50 ms just after an attack and then further increased to 7.32 ± 1.12 ms at 30 min after drip infusion of sodium bicarbonate (100 mg) plus diazepam (5 mg). 3 days after treatment, T5 was 2.37 ± 0.32 msec, which is significantly lower than that just after treatment (*P* < 0.01).

Sympathetic function as measured by electronic pupillometry parameters VD and T5 (Figure 3) showed no difference between the affected and unaffected sides in the baseline data measured in the stable phase (VD: affected side is 31.02 ± 6.16 mm/sec, unaffected side is 29.25 ± 5.73 mm/sec; T5: affected side is 3.37 ± 0.43 msec, unaffected side is 3.25 ± 0.39 msec). In contrast, just before an attack, the sympathetic function on the affected side was significantly activated and then was significantly suppressed after treatment. VC, which represents the parasympathetic function, was markedly suppressed on the affected side just before an attack, which was consistent with the results of the HRV power spectrum analysis (Table 1). This suggests that the sympathetic function on the affected side is relatively overactivated. After treatment, the sympathetic function decreased while the parasympathetic function increased. Subsequently, 3 days after treatment, no difference between affected and unaffected sides was observed in sympathetic function, but parasympathetic function was still significantly suppressed on the affected side. This results in a relatively activated sympathetic function on the affected side even 3 days after an attack.

**Figure 4:**
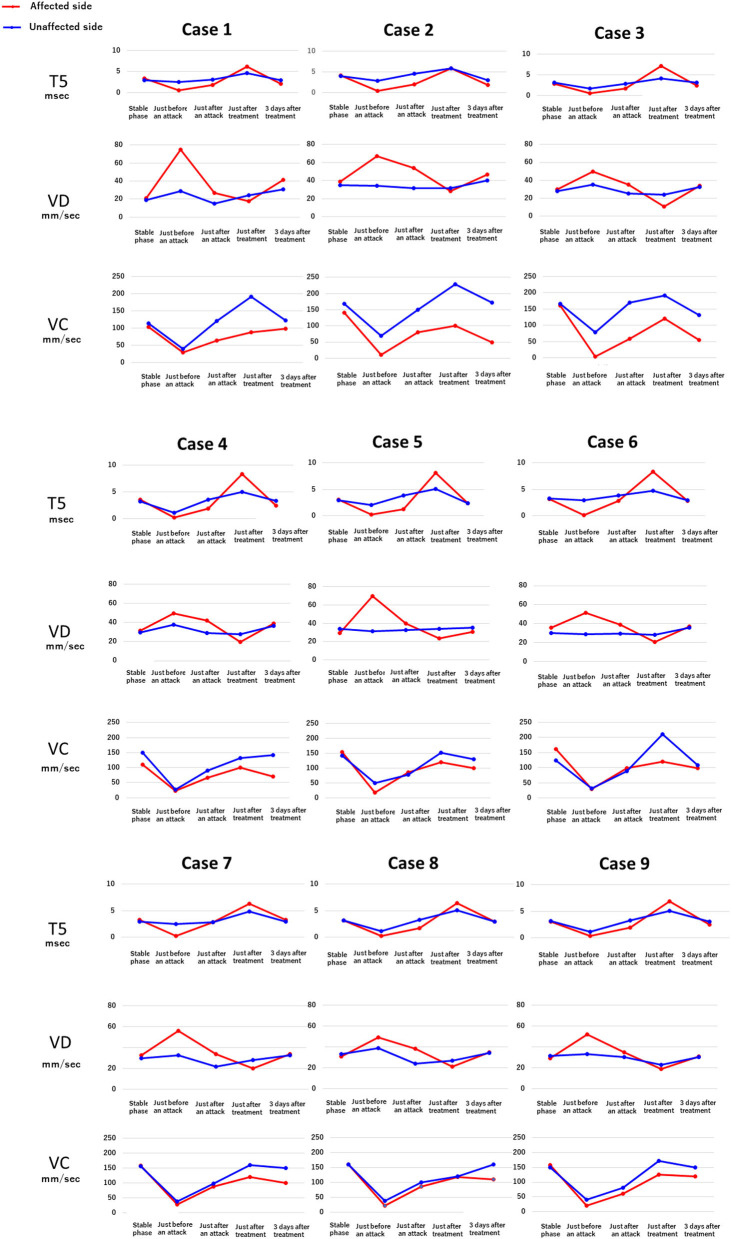
Temporal changes in the electronic pupillometry data and symptoms in nine patients. Immediately before an attack, hyperactivation of the sympathetic nervous system and suppression of the parasympathetic nervous system were found, resulting in relative overactivation of the sympathetic nervous system. Maximum dilation velocity, VD (mm/sec): larger values indicate a more activated the sympathetic nervous system. T5 (msec): smaller values indicate a more activated sympathetic nervous system. Maximum constriction velocity, VC (mm/sec): larger values indicate a more activated parasympathetic nervous system.

Figure 4 shows the results of electronic pupillometry for each of the nine patients with Meniere's disease. All nine patients received the same treatment: an intravenous infusion of 100 mg sodium bicarbonate (NaHCO_3_) plus 5 mg diazepam was administered for its antiemetic effect once just after an attack in the outpatient department. After 30 min of infusion, symptoms such as nausea, vomiting, dizziness, and anxiety were alleviated in all nine patients, and therefore all of them went home without being hospitalized. Each of the nine patients had neck and shoulder stiffness and tension headache (not migraine), among the co-occurring symptoms shown in Table 3, both of which deteriorated immediately before an attack. Eight patients had cold extremities, which was also aggravated immediately before an attack. After the treatment, these symptoms improved in all patients. Six patients had IBS and five patients had GERD, and eight patients had previously or currently had s*himoyake* (chilblains); however, IBS, GERD, and *shimoyake* (chilblains) were not relieved by a single intravenous infusion after an attack. The temporal changes in symptoms in Table 3 were based on the patients' own subjective assessments.

**Table 3:** The treatment after an attack suppressed the hyperactivity of the sympathetic nervous system and restored the suppressed parasympathetic nervous system.

	**CASE 1**					**CASE 2**					**CASE 3**				
**Stiff neck & shoulders**	**+**	**++**	**++**	**-**	**+**	**+**	**++**	**++**	**-**	**+**	**+**	**++**	**++**	**-**	**+**
**Tension headache**	**+**	**++**	**++**	**-**	**-**	**+**	**++**	**++**	**-**	**-**	**+**	**++**	**++**	**-**	**-**
**Cold extremities**	**+**	**++**	**++**	**-**	**-**	**+**	**++**	**++**	**-**	**+**	**+**	**++**	**++**	**-**	**+**
**IBS**	**+**	**+**	**+**	**+**	**+**	**+**	**+**	**+**	**+**	**+**	**+**	**+**	**+**	**+**	**+**
**GERD**	**+**	**+**	**+**	**+**	**+**	**+**	**+**	**+**	**+**	**+**	**+**	**+**	**+**	**+**	**+**
	**Stable phase**	**Just before an attack**	**Just after an attack**	**Just after treat-ment**	**3 days after treat-ment**	**Stable phase**	**Just before an attack**	**Just after an attack**	**Just after treat-ment**	**3 days after treat-ment**	**Stable**	**Just before an attack**	**Just after an attack**	**Just after treat-ment**	**3 days after treat-ment**
	**CASE 4**					**CASE 5**					**CASE 6**				
**Stiff neck & shoulders**	**+**	**++**	**++**	**-**	**+**	**+**	**++**	**++**	**-**	**+**	**+**	**++**	**++**	**-**	**-**
**Tension headache**	**+**	**++**	**++**	**-**	**+**	**+**	**++**	**++**	**-**	**+**	**+**	**++**	**++**	**-**	**-**
**Cold extremities**	**+**	**++**	**++**	**-**	**+**	**+**	**++**	**++**	**-**	**+**	**+**	**++**	**++**	**-**	**-**
**IBS**	**+**	**+**	**+**	**+**	**+**	**+**	**+**	**+**	**+**	**+**	**-**	**-**	**-**	**-**	**-**
**GERD**	**-**	**-**	**-**	**-**	**-**	**+**	**+**	**+**	**+**	**+**	**-**	**-**	**-**	**-**	**-**
	**Stable phase**	**Just before an attack**	**Just after an attack**	**Just after treat-ment**	**3 days after treat-ment**	**Stable phase**	**Just before an attack**	**Just after an attack**	**Just after treat-ment**	**+**	**Stable phase**	**Just before an attack**	**Just after an attack**	**Just after treat-ment**	**3 days after treat-ment**
	**CASE 7**					**CASE 8**					**CASE 9**				
**Stiff neck & shoulders**	**+**	**++**	**++**	**-**	**+**	**+**	**++**	**++**	**-**	**-**	**+**	**++**	**++**	**-**	**+**
**Tension headache**	**+**	**++**	**++**	**-**	**-**	**+**	**++**	**++**	**-**	**-**	**+**	**++**	**++**	**-**	**-**
**Cold extremities**	**+**	**++**	**++**	**-**	**-**	**-**	**-**	**-**	**-**	**-**	**+**	**++**	**++**	**-**	**-**
**IBS**	**+**	**+**	**+**	**+**	**+**	**-**	**-**	**-**	**-**	**-**	**-**	**-**	**-**	**-**	**-**
**GERD**	**-**	**-**	**-**	**-**	**-**	**+**	**+**	**+**	**+**	**+**	**-**	**-**	**-**	**-**	**-**
	**Stable phase**	**Just before an attack**	**Just after an attack**	**Just after treat-ment**	**3 days after treat- ment**	**Stable phase**	**Just before an attack**	**Just after an attack**	**Just after treat-ment**	**3 days after treat-ment**	**Stable phase**	**Just before an attack**	**Just after an attack**	**Just after treat-ment**	**3 days after treat-ment**

*As a result, neck and shoulder stiffness, tension headache, and cold extremities induced by the tense sympathetic nervous system were improved. Because of the chronic nature of IBS and GERD, they were not improved by a single intravenous infusion*.

*–, no symptoms; +, symptoms; ++, severe symptoms by the patients' own subjective assessment*.

## Discussion

The use of methacholine, as cholinergic ophthalmic drops (parasympathomimetic drug), in patients with Ménière's disease demonstrated that there was a difference between the affected and unaffected sides, with the affected side showing abnormal parasympathetic function (18). The methacholine test is a method to observe the increase in cholinergic hyperreactivity of the pupillary reaction by the use of methacholine in patients with parasympathetic hypofunction. Studies by electronic pupillometry using methacholine ophthalmic drops were conducted in the 1980s and 1990s. A study using methacholine to examine pupillary reaction in patients with Ménière's disease showed cholinergic hyperreactivity (9).

However, the interpretation of that report requires careful consideration. Neural disinhibition occurs when there is parasympathetic hypofunction, and parasympathomimetic agents, such as methacholine, increase iris hypersensitivity, resulting in excessive contraction of the iris (15); thus, cholinergic hyperreactivity observed with the methacholine test is a finding that indirectly suggests parasympathetic hypofunction.

Problems with methacholine have recently been found, and therefore, this drug is currently not used for clinical studies of autonomic nervous function. This is because there are considerable interindividual differences in response to the concentrations of the drops. In addition, it is difficult to determine the optimal concentration. Overreaction to high concentrations may cause cholinergic hyperfunction (19). Such interindividual differences can occur also in people wearing contact lenses, those at older ages, and those with diabetes mellitus; parasympathetic hypofunction should therefore be cautiously analyzed (15). Light reflex is double innervated by sympathetic and parasympathetic nerves; the sympathetic response cannot be analyzed correctly when parasympathomimetic drugs are used.

The electronic pupillometry was performed using a CMOS sensor with five million pixels for image analysis, which has been greatly improved over the past 30 years. It is small and light with dedicated LEDs for infrared illumination for monitoring pupil diameter and white light flashes to stimulate pupil constriction. We used an iOS app as analysis software. When the latest iPad or iPhone is used, the high-speed image processing system enables us to instantly analyze the autonomic function of sympathetic and parasympathetic nerves without using any drugs. Furthermore, results can be obtained in real time, which greatly reduces the burden on patients.

Our present study successfully measured both sympathetic and parasympathetic activities simultaneously immediately before Ménière's attacks by using HRV and the latest electronic pupillometry. As a result, suppression of parasympathetic activity and excitation of sympathetic activity (relatively further over excitation of sympathetic activity) were found. Our result of parasympathetic suppression was consistent with the results of previous studies using the methacholine test.

Autonomic activity is generally explained by the balance between the sympathetic and parasympathetic nerves distributed throughout the body. Given that autonomic activity is affected by stress, the interaction between the central and autonomic nervous systems is important, and recently, this interaction has been proposed to be via the central autonomic network (CAN) (20, 21). The CAN is a central network including the limbic system with the amygdala and the hippocampus that surrounds the hypothalamus, the prefrontal cortex anteriorly, and the solitary nucleus in the medulla inferiorly (21). The CAN affects blood pressure, circulatory regulation, body temperature regulation, hormonal metabolism, and digestion and excretion. It is also greatly involved in the HRV measured in this study; power spectrum analysis of HRV can thus serve as an objective indicator of stress (22). Over activity of the CAN suppresses parasympathetic function and activates sympathetic function.

As described above (Figure 2), *shimoyake* (chilblains) is a frequent symptom in patients with Ménière's disease. *Shimoyake* (chilblains) is a condition with reddish swelling on the acral parts of the body, such as the tips of the fingers or toes, accompanied by pain and itching during the winter and early spring (23). Capillary permeability, edema, and inflammation around vessels are crucially involved in the pathogenesis of *shimoyake* (chilblains) (24). There are two types of capillaries in the tips of the fingers and toes. In type A, an arteriole simply branches into capillaries and then the capillaries coalesce into a venule, whereas in type B, arteriovenous anastomoses (AVAs), which connect an arteriole and a venule, are present in addition to the simple structure of type A. Both type A and type B capillaries are present in the stria vascularis (25); however, only type A capillaries are present in regions around spiral ganglion cells, vestibular dark cells, and vestibular ganglion cells (25).

D-IBS, often seen in patients with Ménière's disease (Figure 2), is also associated with overactivation of sympathetic nerves and secretion of corticotropin releasing factor from the central nervous system, reducing the reabsorption of water in the small intestine and increasing the vascular permeability of the large intestine (26), which may have a genetic component (27). The pain of IBS also overactivates CAN (20, 21), leading to overactivation of the sympathetic nervous system; in consequence, activated TRPV4 further increases vascular permeability (28, 29). A study of patients with Ménière's disease has demonstrated increased intestinal vascular permeability in patients with active disease compared with those with no vertigo spells for at least 3 months (30).

Sodium bicarbonate (NaHCO_3_) has the effect of dilating blood vessels, which can reduce the tension of blood vessels, resulting in increased blood flow during an attack induced by sympathetic hyperactivity (31). An intravenous infusion of NaHCO_3_ also has the effect of suppressing excitation of the vestibular nuclei (32). Diazepam, a type of benzodiazepine, is often used to treat Ménière's attacks (8). Diazepam suppresses the activity of the central nervous system. Our study suggests that diazepam also suppresses over activity of the CAN, resulting in the alleviation of autonomic symptoms, such as nausea and vomiting, and relieves anxiety owing to its antianxiety effect. All nine patients usually experienced repeated nausea and vomiting for several hours after the onset of an attack. (The mean duration of an attack in patients without treatment was 3.5 h [1.5–6 h]). However, all nine patients recovered from the symptoms in a short time by this treatment. Therefore, we considered this treatment effective in improving the symptoms. In addition, all patients with stiff neck and shoulders, cold extremities, and tension headache improved after treatment. These improvements are also thought to be the result of relaxing sympathetic tone and reduced tension and increased blood flow.

The sympathetic hyperactivity and parasympathetic suppression that occurred immediately before an attack were reversed by the post-attack treatment, and it is considered that the neck and shoulder stiffness, tension headache, and cold extremities induced by the tension of the sympathetic nervous system were also improved by this treatment. Because of the chronic nature of IBS and GERD, they were not improved by a single intravenous infusion.

We prescribed only betahistine (8) as an interval therapy, and did not prescribe any saluretics or other diuretics. We also prescribed promethazine as a prophylactic for all patients. We instructed the patients to control their condition by themselves by taking promethazine (8) orally as needed when they felt that symptoms were imminent or when an attack occurred.

Renal disease and inner ear disease have something in common: aminoglycoside nephrotoxicity can also cause inner ear disorders (33). TRPV4 is localized in collecting duct cells of the kidney (34). TRPV4 overactivated by aminoglycoside causes pathological activation of Ca^++^ channels, leading to excess efflux of K^+^ which damages the collecting duct cells, resulting in renal dysfunction (35). Aminoglycoside also abnormally activates TRPV4 in the lumen and periphery of the stria vascularis, which causes excess efflux of K^+^, resulting in inner ear dysfunction associated with cell degeneration (35).

Assuming that continuous excessive stress causes sympathetic hyperactivity resulting, as with the pathogenesis of *shimoyake* (chilblains), then an abnormal influx of K^+^ can be assumed to occur in the inner ear. More specifically, abnormal influx of K^+^ from the stria vascularis into the endolymph causes hearing impairment, and abnormal influx of K^+^ from capillaries around the spiral ganglion cells, vestibular dark cells, and vestibular ganglion cells into the perilymph induces irritative nystagmus (36), followed by paralytic nystagmus (37).

Sympathetic nerves are distributed in the arterioles of the inner ear (38). In addition, adrenergic receptors are also present in the inner ear (39, 40) and these receptors are stimulated in conjunction with sympathetic nerves to achieve a balance of K^+^ cycling between K^+^ secretion into endolymph and K^+^ efflux from endolymph (41). Under conditions of an activated sympathetic nervous system, arterioles usually do not constrict continuously due to vasorelaxation factors under homeostasis. However, the present study revealed the overexcitation of the sympathetic nervous system just before an attack of Ménière's disease. Such conditions would induce shear stress in vascular epithelial cells of the capillaries of the stria vascularis and around the spiral and vestibular ganglion cells, resulting in failure in the balance of vascular tone. As a result, vascular permeability would increase, as is the case with *shimoyake* (chilblains) on the tips of the fingers and toes.

In the stria vascularis, AVAs between arterioles and venules are widely distributed in the cochlea, as with in the tips of the fingers and toes, from the apical region to the hook region (25). In a study that examined histological specimens taken from people with normal hearing and from those with Ménière's disease, no histological difference was found in outer sulcus cells of the upper apical region, which are involved in low-tone hearing loss in Ménière's disease; however, a difference was found in outer sulcus cells of the middle and basal turns (42). The basal membrane of the upper apical region is thinner and has an area larger than that of other parts; therefore, the upper apical region is more susceptible than other parts to endolymphatic pressure suppressing auditory vibrations. In addition, the capillaries of the stria vascularis are more densely distributed in the upper apical region than in other parts (25), and thus there is more likely to be an excess of K^+^ in the upper apical region than in other parts. These factors may impair neuronal function in hearing to cause low-tone hearing loss in Ménière's disease.

From the relationship between pernio and inflammation, coldness causes hyperactivity of the sympathetic nervous system, resulting in increasing vascular permeability, edema, and perivascular inflammation of the fingertips. Under such conditions, monocytes, macrophages, IL-6, IL-1β, and TNF-α, which are involved in increasing oxidative stress and peripheral vascular inflammation, are likely to enhance subsequent progression of inflammation (43). Macrophages (inflammatory monocytes) produce inducible nitric oxide synthase (iNOS); therefore, stress induced by persistent coldness creates a vicious cycle of inflammation. There are individual differences in phenotypes of these reactions. In Ménière's disease, continuous overstress also causes the sympathetic hyperactivity to increase vascular permeability and perivascular inflammation to develop in the inner ear, as is the case with pernio. This enhances oxidative stress to create a vicious cycle of inflammation, resulting in progressive and chronic neuroinflammation.

A study using 3-tesla delayed post-contrast MR imaging noted increased vascular permeability of the blood–perilymph barrier in the cochlea of patients with Ménière's disease and significantly increased permeability compared with patients with sudden sensorineural hearing loss (44). The diagnostic imaging in that study revealed a close association of Ménière's disease with vascular permeability, as with pernio. The blood–labyrinthine barrier in the normal human inner ear is similar to the blood–brain barrier (BBB): it is composed of vascular endothelial cells joined by tight junctions and is surrounded by pericytes and an intact basement membrane (45). Overactivity of the CAN is known to be associated with oxidative stress, triggering damage. Immunohistochemical studies of the microvasculature of the utricular macula from patients with intractable Ménière's disease demonstrate excessive iNOS and nitrotyrosine, which is indicative of oxidative damage (46). Furthermore, with recurrent overactivation of the sympathetic nervous system, one might expect damage and functional and structural changes to the microvasculature of the inner ear (47).

Further detailed studies should be conducted on the structure and function of AVAs from the apical region to the hook region of the cochlea and of capillaries in the otolithic organs and the semicircular canal ampulla as well as on the vasorelaxation factors associated with TRP channels in the vascular endothelial cells and vascular permeability.

## Data Availability Statement

The original contributions presented in the study are included in the article/supplementary material, further inquiries can be directed to the corresponding author/s.

## Ethics Statement

The studies involving human participants were reviewed and approved by the study was reviewed and approved by the Ethics Committee of JCHO Tokyo Shinjuku Medical Center (R3-7). The patients/participants provided their written informed consent to participate in this study.

## Author Contributions

MI initiated and designed the study, and collected data. MI, YK, and FM conducted data analysis and MI wrote the main manuscript text. GI, AI, YK, FM, and YI reviewed the manuscript. All authors contributed to the article and approved the submitted version.

## Conflict of Interest

The authors declare that the research was conducted in the absence of any commercial or financial relationships that could be construed as a potential conflict of interest.

## Publisher's Note

All claims expressed in this article are solely those of the authors and do not necessarily represent those of their affiliated organizations, or those of the publisher, the editors and the reviewers. Any product that may be evaluated in this article, or claim that may be made by its manufacturer, is not guaranteed or endorsed by the publisher.

## Abbreviations

ANOVA: analysis of variance
AVA: arteriovenous anastomosis
BBB: blood–brain barrier
CAN: central autonomic network
D-IBS: diarrhea-predominant irritable bowel syndrome
GERD: gastro-esophageal reflux disease
HRV: heart rate variability
iNOS: inducible nitric oxide synthase
NaHCO_3_: sodium bicarbonate
T5: time to 63% redilation
TRPV4: transient receptor potential vanilloid 4
VC: maximum constriction velocity
VD: maximum dilation velocity.
